# Uterine tumors mimicking ovarian sex cord tumors with rhabdoid differentiation: a clinicopathologic study of 4 cases: A case series analysis

**DOI:** 10.1097/MD.0000000000039123

**Published:** 2024-08-16

**Authors:** Hongling Li, Le Xie, Jinhui Zhang, Yuanyuan Xu, Xingyan Wu, Zengwei Chen, Rongjun Mao

**Affiliations:** a Department of Pathology, Foshan Hospital of Traditional Chinese Medicine, Guangzhou University of Chinese Medicine, Foshan, China.

**Keywords:** ESR1, GREB1, NCOA2/3, prognosis, rhabdoid cell, uterine tumors resembling ovarian sex cord tumors

## Abstract

**Rationale:**

Uterine tumors resembling ovarian sex cord tumors (UTROSCT) with rhabdoid features are uncommon mesenchymal neoplasms exhibiting diverse histological patterns, including significant rhabdoid morphology. A thorough comprehension of their clinicopathologic features is crucial for precise diagnosis and effective management.

**Patient concerns:**

This study presents 4 cases of UTROSCT with rhabdoid features, diagnosed in patients aged 31 to 58. Varied recurrence patterns were observed, including similar recurrent lesions to the primary tumors with subsequent mortality, initial invasion and lymph node metastasis, and presence of only primary tumor.

**Diagnoses:**

Histopathological examination revealed diverse morphological patterns, prominently featuring rhabdoid differentiation. Immunohistochemical analysis showed expression of hormone receptors, sex cord, smooth muscle, and epithelial markers, notably WT1, CD56, and CD99. Molecular analysis identified ESR1-NCOA2 fusions and ESR1 and NCOA2/3 rearrangements, indicating a potential association between these genetic alterations and extensive rhabdoid differentiation.

**Interventions:**

Various treatments were administered post-recurrence, including chemotherapy and targeted therapies. However, poor clinical outcomes were observed in all cases.

**Outcomes:**

Despite aggressive treatments, including chemotherapy and targeted therapies, poor clinical outcomes were observed, highlighting the aggressive nature of UTROSCT with significant rhabdoid differentiation.

**Lessons:**

This case series emphasizes the importance of detailed pathological reporting, comprehensive molecular testing, and thorough tumor staging in UTROSCT cases with rhabdoid features. Enhanced understanding of the clinicopathologic characteristics of UTROSCT with rhabdoid differentiation is crucial for accurate diagnosis, prognostication, and management strategies.

## 1. Introduction

Uterine tumors resembling ovarian sex cord tumors (UTROSCT) are rare mesenchymal tumors, recently recognized by the latest edition of the World Health Organization classification as a uterine neoplasm with morphological patterns resembling those seen in ovarian sex cord tumors, lacking a recognizable component of endometrial stroma.^[1]^ Histologically, they primarily exhibit sex cord-like differentiation and demonstrate variable immunoreactivity for sex cord markers (inhibin, CR, WT1, CD56, CD99, SF1, FOXL2, Melan-A), epithelial markers, estrogen receptor (ER), progesterone receptor (PR), smooth muscle markers (actin, desmin, H-caldesmon), and CD10. In most cases, UTROSCT is benign, but it should be considered to have low malignant potential due to the possibility of recurrence or metastasis, some patients have succumbed to the disease.^[1–4]^ Due to the rarity and morphological diversity of UTROSCT, identifying prognostic features presents a challenge. Recent studies have investigated various clinical and pathological indicators that could correlate with UTROSCT prognosis, including age, tumor size, degree of sex cord differentiation, mitotic activity, necrosis, presence of infiltrative margins and cervical involvement, involvement of other sites, cellular atypia, vascular invasion, expression of sex cord markers, and fusion genes.^[1,5–7]^ In the largest follow-up series reported,^[1]^ one of the 5 cases of malignant UTROSCT exhibited extensive rhabdoid morphology, a feature often associated with aggressive behavior and adverse outcomes.^[2–4,8–15]^ Researchers including Watrowski et al^[16]^ reported 2 new cases of UTROSCT. They found that UTROSCT with GREB1::NCOA2 gene fusion or PD-L1 molecular expression appears to be prone to metastasis and recurrence. They advocate for the subdivision of UTROSCT based on molecular criteria.^[16]^ We have collected 4 cases of UTROSCT with rhabdoid features, all of which retained expression for SMARCB1, SMARCA2, or SMARCA4. This report investigates the clinical, morphological, immunohistochemical, and molecular characteristics, as well as patient outcomes, of UTROSCT with rhabdoid features.

## 2. Materials and methods

### 2.1. Case selection

Recently, we encountered 4 cases of UTROSCT with rhabdoid differentiation during pathological consultations at Foshan Traditional Chinese Medicine Hospital. Among these cases, 3 exhibited significant rhabdoid cell presence, while 1 case demonstrated approximately 30% rhabdoid morphology. Clinical data and follow-up information were retrieved from medical records. A review of hematoxylin and eosin staining and immunohistochemical slides of the primary tumors (4 cases) and recurrences (2 cases) was conducted. The panel of immunohistochemical stains included inhibin, calretinin, ER, PR, WT1, CAM5.2, desmin, SMA, H-caldesmon, CD56, CD99, CD10, SMARCB1, SMARCA2, SMARCA4, SF1, EMA, AE1/AE3, and Ki67 in all tumors. Stains were interpreted as negative, focally positive (<50% staining), or diffusely positive (≥50% staining).

### 2.2. Fluorescence in situ hybridization

Fluorescence in situ hybridization (FISH) analysis was conducted on 4 μm FFPE slides using break-apart probes for GREB1, ESR1, NCOA2, and NCOA3 (LBP Medicine Science & Technology Co., Ltd., Guangzhou, China) to detect rearrangements of the respective genes. Standard protocols were followed in our laboratory, where at least 50 nonoverlapping nuclei were counted, ensuring that at least 15% of tumor cells exhibited split patterns and/or single signals. In cases where results approached the cutoff values, additional nuclei were counted or alternative counting methods were employed.

### 2.3. Next-generation sequencing

Cases 1 and 2 underwent next-generation sequencing (NGS) based on DNA/RNA at OrigiMed Technology Inc., Shanghai, China. DNA extraction was carried out using the QIAamp DNA FFPE Tissue Kit (Qiagen, CA) and quantified with the Qubit dsDNA HS Assay Kit (Life Technologies, Carlsbad, CA). The gDNA library was captured using a customized 671 gene individually-synthesized 5’-biotinylated DNA 120 bp oligonucleotide probe panel with xGen Hybridization and then quantified again with the Qubit dsDNA HS Assay Kit. RNA extraction utilized the miRNeasy FFPE Kit (Qiagen, Hilden, Germany) and quantification was performed using the Qubit RNA HS Assay Kit (Thermo Fisher, Waltham, MA). The cDNA library was captured with a customized 632 gene individually synthesized 5’-biotinylated DNA 120 bp oligonucleotide probe panel with xGen Hybridization and Wash Kit (IDT, Coralville, IA). Subsequently, the captured libraries were sequenced on Illumina NovaSeq 6000 with 2 × 150 bp paired-end reads, following the manufacturer’s instructions (Illumina, San Diego, CA). A comprehensive genomic analysis of the samples was performed with a DNA + RNA cancer-related gene panel (YuanSu S, 671 DNA and 632 RNA gene panel; OrigiMed, Shanghai, China). DNA-seq reads were mapped to the hg19 reference sequence using BWA (version 0.7.12). PCR duplicates were removed with Pi-card (version 2.5.0), and recalibration was conducted using the BaseRecalibrator tool from GATK (version 3.1.1). A proprietary in-house-developed algorithm was employed for DNA fusion detection. For RNA-seq, reads were mapped using the STAR algorithm (version 2.5.3) and STAR-fusion (version 0.8) was utilized for fusion detection.^[17]^ Gene fusions were identified when the total number of supportive reads spanning the fusion junction was ≥5.^[18]^

## 3. Results

### 3.1. Clinical characteristics

#### 3.1.1. Case 1

##### 3.1.1.1. Clinical presentation

A 31-year-old female underwent laparoscopic myomectomy for a presumed uterine leiomyoma. Pathological examination revealed a uterine tumor resembling an ovarian sex cord tumor, prompting subsequent total hysterectomy via laparoscopy.

##### 3.1.1.2. Follow-up and recurrence

She underwent regular follow-ups without adjuvant therapy. Eight years later, the first recurrence occurred in the pelvic-abdominal cavity, followed by multiple recurrences with metastases to the liver, lungs, and adrenal glands.

##### 3.1.1.3. Treatment and outcome

She underwent several surgeries for recurrent tumors and surrounding tissue excision. After recurrence, she received medical castration therapy, oral megestrol, goserelin, letrozole treatments, multiple tumor ablation procedures, paclitaxel plus cisplatin chemotherapy, and tislelizumab immunotherapy. However, the tumor progressed continuously, and she passed away 6 years after the first recurrence.

#### 3.1.2. Case 2

##### 3.1.2.1. Clinical presentation

A 58-year-old female presented with right leg swelling and was found to have multiple heterogeneous enhancing masses in the pelvis on CT.

##### 3.1.2.2. Surgical findings

A hysterectomy with bilateral adnexectomy was performed. A woven-like tumor mass measuring 8 cm × 4.5 cm × 4 cm was identified in the myometrium, with several nodular neoplasms of varying sizes visible on the serosal surface. Extensive tumor invasion was noted in the uterine body and cervix, with tumor metastasis observed in the bilateral adnexa, pelvic wall, and 7 pelvic lymph nodes.

##### 3.1.2.3. Treatment and outcome

Initially diagnosed as leiomyosarcoma by another hospital, she underwent 6 cycles of chemotherapy with various regimens: doxorubicin plus albumin plus paclitaxel, albumin plus paclitaxel plus bevacizumab, gemcitabine plus dacarbazine plus anlotinib, gemcitabine plus dacarbazine, and pamiparib plus anlotinib. Currently, she is 12 months postoperative with fair general condition.

#### 3.1.3. Case 3

##### 3.1.3.1. Clinical presentation

A 31-year-old female underwent myomectomy in 2012 after a uterine leiomyoma was discovered during a routine health checkup. The tumor was assessed at multiple institutions, resulting in varying diagnoses, including low-grade endometrial stromal sarcoma, undifferentiated endometrial stromal sarcoma, and high-grade endometrial stromal sarcoma.

##### 3.1.3.2. Treatment and recurrence

She underwent 4 cycles of chemotherapy with ifosfamide, cisplatin, and doxorubicin hydrochloride. Four years later, recurrence in the uterus with pulmonary metastasis occurred, leading to 1 cycle of chemotherapy with ifosfamide, nedaplatin, and doxorubicin, followed by hysterectomy with bilateral adnexectomy and pelvic and abdominal aortic lymph node dissection. She received megestrol for 5 months plus 6 injections of enantone, but disease progression persisted, necessitating lung tumor ablation surgery.

##### 3.1.3.3. Outcome

Seven years after the initial diagnosis, significant pleural effusion developed. She underwent oral keytruda administration 4 times plus anlotinib. The lung metastasis biopsy showed morphology consistent with the primary uterine tumor. Treatment continued with keytruda plus bevacizumab for 6 cycles, oral letrozole plus 4 cycles of chemotherapy (gemcitabine plus docetaxel), but disease control remained elusive. She passed away 6 years after the first recurrence.

#### 3.1.4. Case 4

##### 3.1.4.1. Clinical presentation

A 49-year-old female underwent hysterectomy with bilateral adnexectomy for an approximately 11 cm uterine leiomyoma.

##### 3.1.4.2. Surgical findings

The tumor was located within the myometrium, with no metastatic lesions or enlarged lymph nodes in the pelvic-abdominal cavity. Initially diagnosed as uterine myxoid leiomyosarcoma by another hospital, no tumor necrosis was observed, and mitotic count was 3 per 10 HPF.

##### 3.1.4.3. Treatment and outcome

She received 8 cycles of chemotherapy (regimen unspecified). Currently, she is 6 months postoperative without recurrence or metastasis.

#### 3.1.5. Pathological features

**Figure 1.**
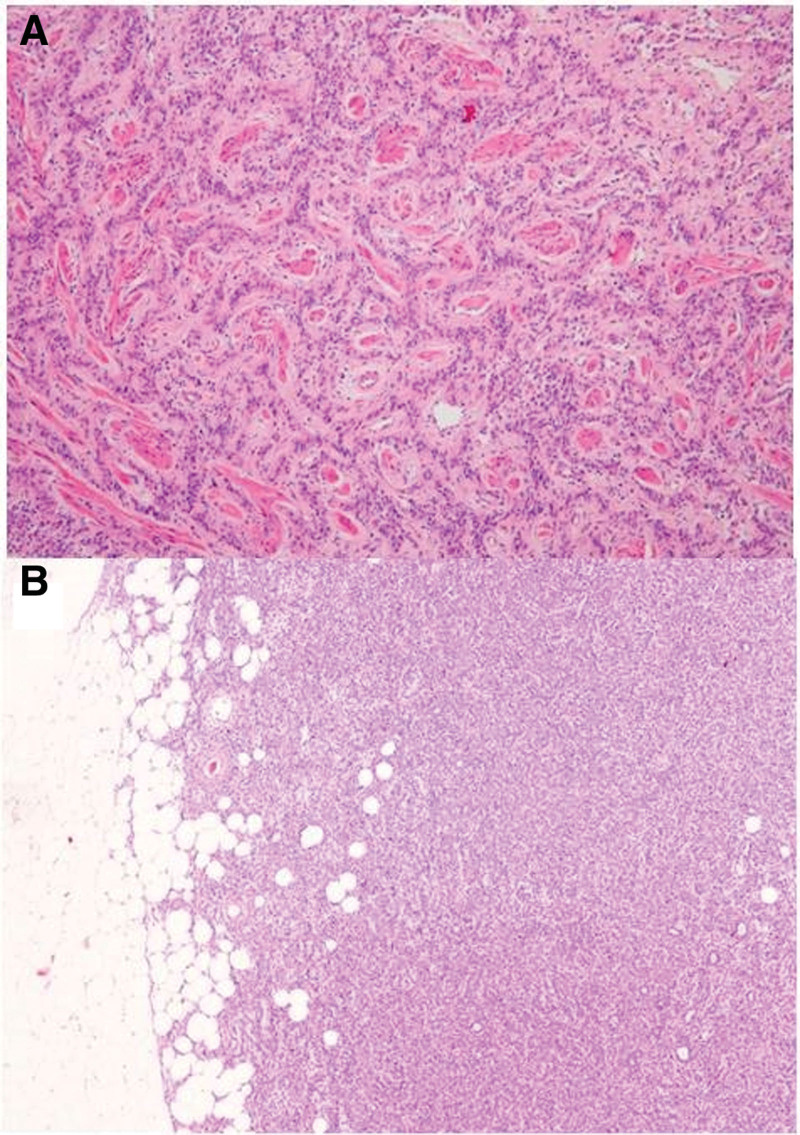
Infiltrative growth into the myometrium (A), extension to paramerial tissue (B).

The primary location of the tumor in all 4 cases was within the myometrium, exhibiting significant infiltrative growth into the surrounding tissues (Fig. 1).

##### 3.1.5.1. Case 1: tumor morphology

**Figure 2.**
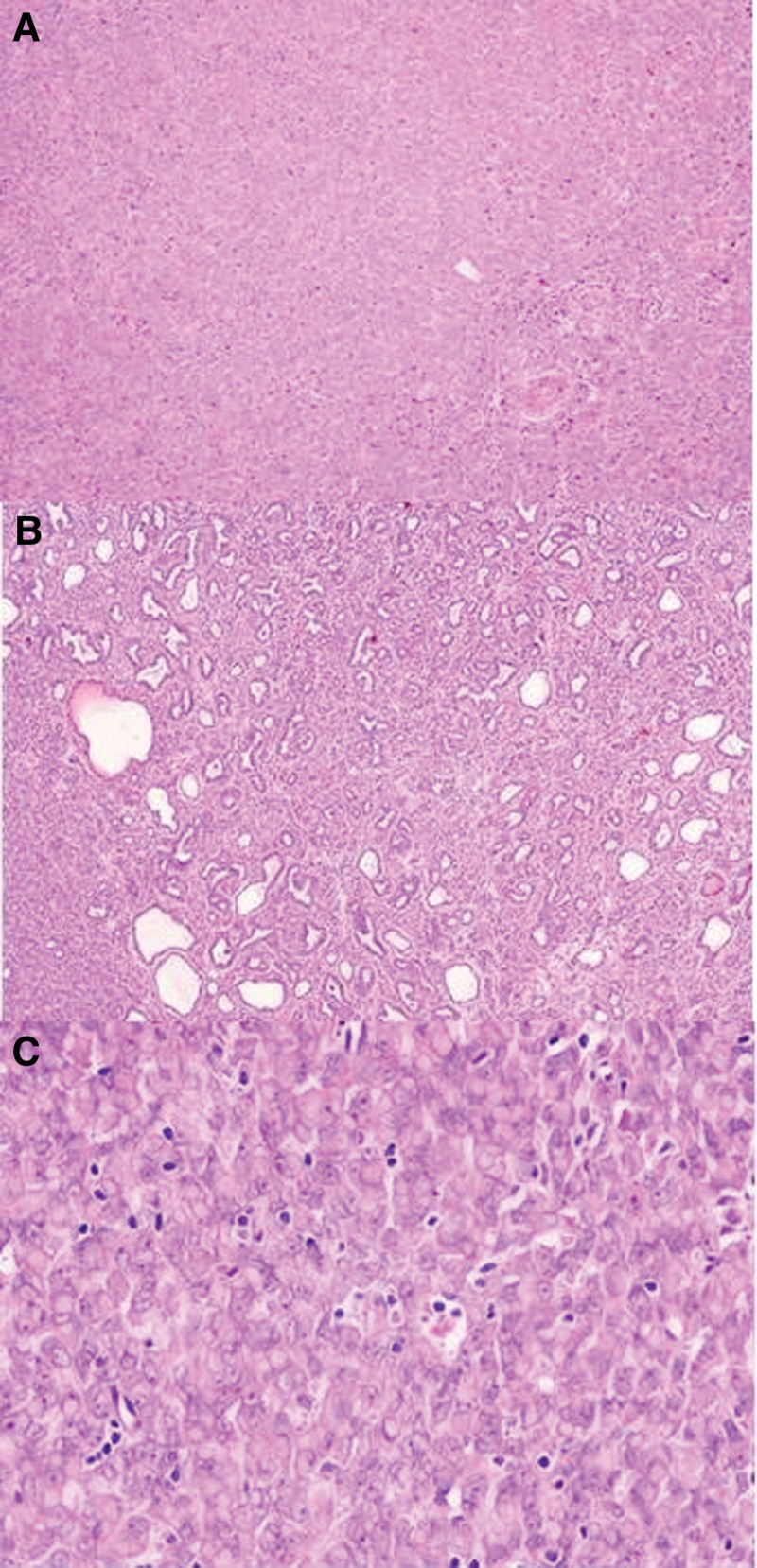
Diffuse (A) and trabecular (B), cord-like (B), pseudoglandular growth (B), rhabdoid tumor cells are prominent (C).

The majority of the tumor tissue exhibited a diffuse, solid sheet-like appearance with trabecular, cord-like, pseudoglandular (Fig. 2A and B), or nested arrangements. Tumor cells displayed eccentric nuclei, prominent nucleoli, and abundant cytoplasm with notable rhabdoid features (Fig. 2C).

##### 3.1.5.2. Mitotic activity and necrosis

Mitoses were rare, and no necrosis or vascular and nerve invasion were observed. In metastatic sites, only a few tumor cells exhibited rhabdoid characteristics.

##### 3.1.5.3. Case 2: tumor morphology

**Figure 3.**
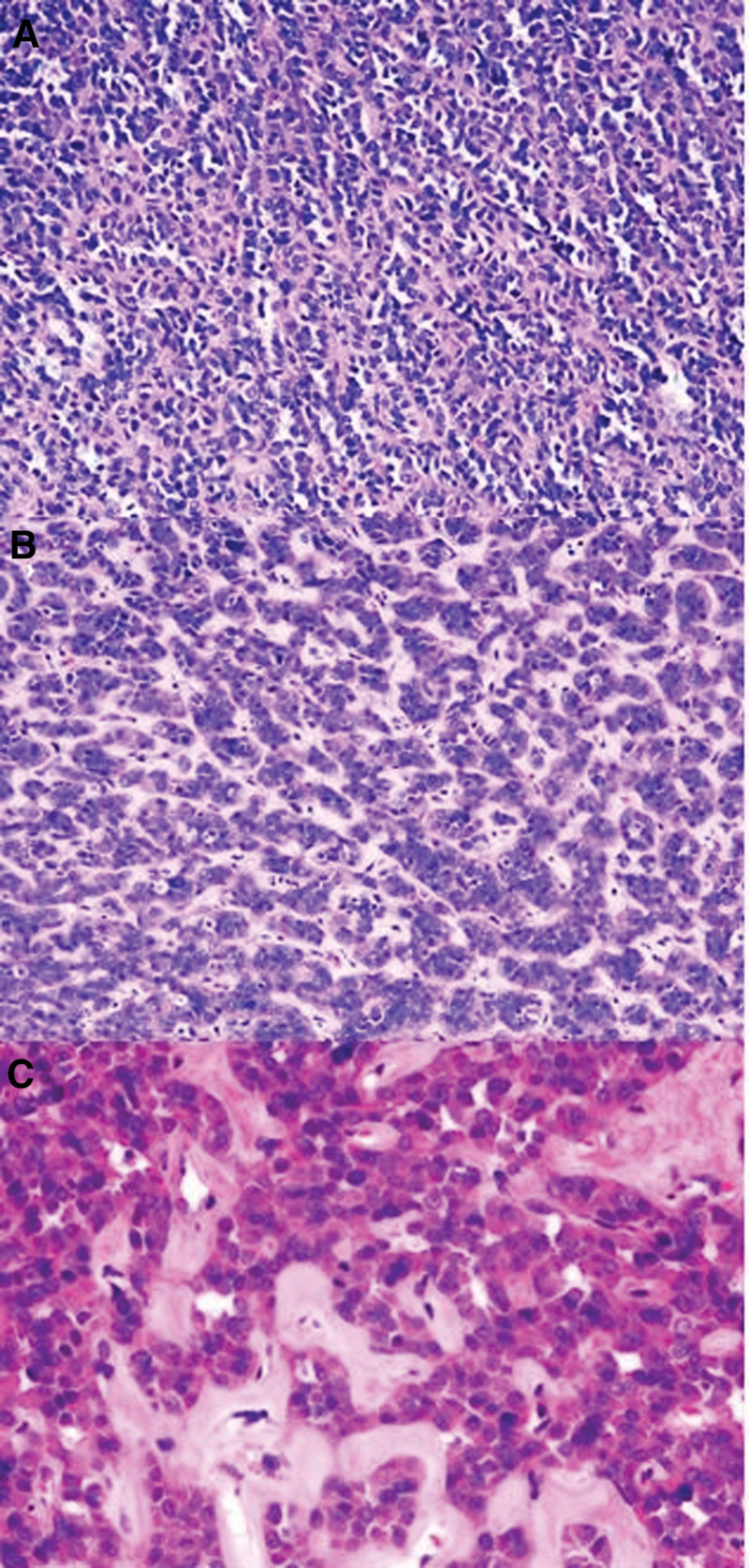
Tubular (A) and trabecular (B) growth patterns, mitoses (C).

The tumor tissue displayed tubular and trabecular patterns (Fig. 3A and B). Tumor cells varied, showing either scant or abundant cytoplasm. Certain areas exhibited cytoplasmic eosinophilia and nuclear displacement, forming rhabdoid morphology in more than 50% of the cells.

##### 3.1.5.4. Mitotic activity and necrosis

Mitotic figures (Fig. 3C) were present, with approximately 4 mitoses per 10 high-power fields (HPF). Focal necrosis was also observed.

##### 3.1.5.5. Case 3: tumor morphology

**Figure 4.**
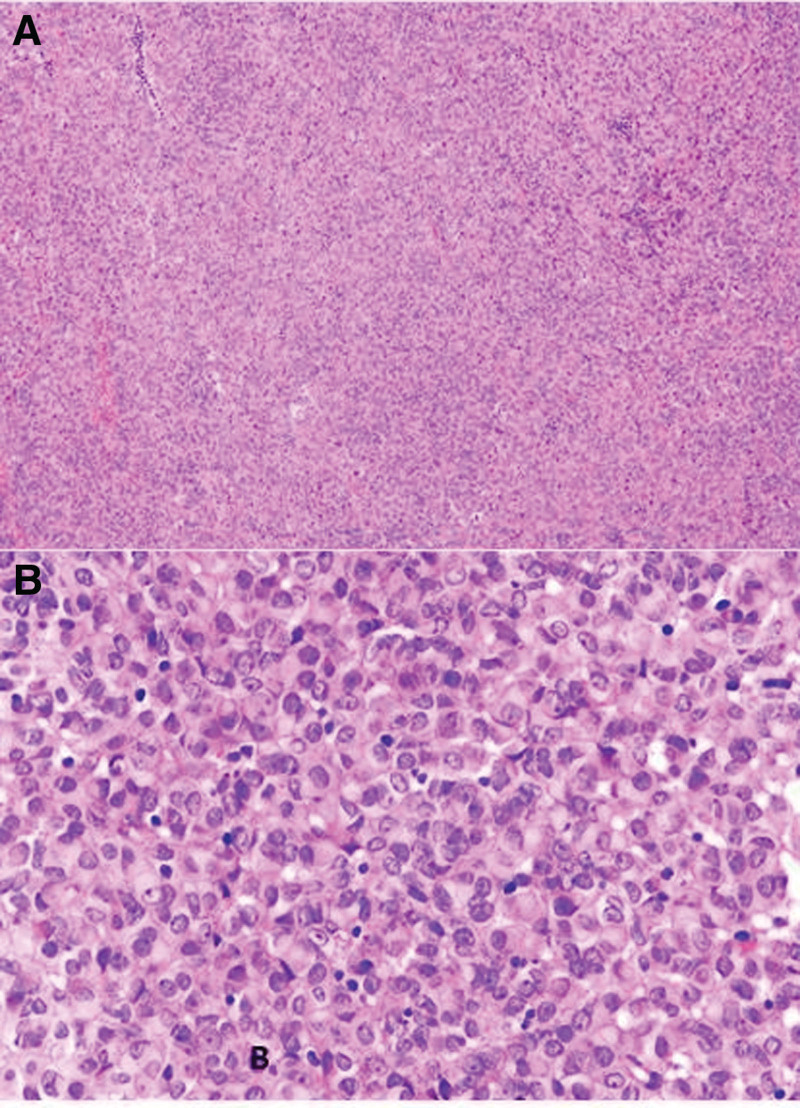
Predominantly diffuse sheet-like growth pattern (A); epithelioid cells with rhabdoid features are striking (B).

The diffuse structure was predominant, with epithelioid cells distributed in sheet-like patterns (Fig. 4A). The tumor exhibited diffuse rhabdoid morphology (Fig. 4B).

##### 3.1.5.6. Mitotic activity and necrosis

Approximately 3 mitoses per 10 HPF were noted, with no necrosis observed.

##### 3.1.5.7. Case 4: tumor morphology

**Figure 5.**
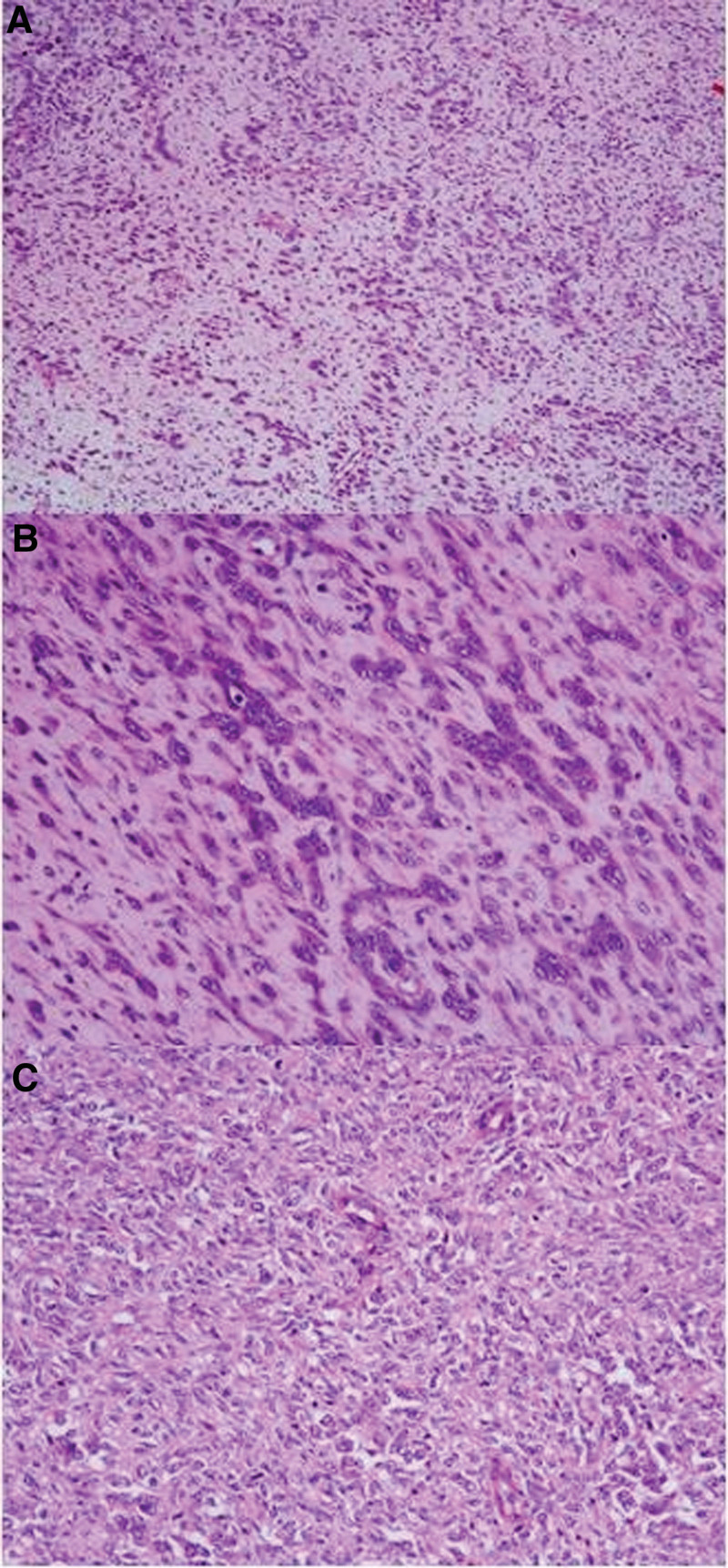
A myxoid background (A). The cells are rhabdoid (B) and spindled (C).

The tumor exhibited a prominent mucinous background (Fig. 5A), with the tissue diffusely or irregularly arranged, lacking typical glandular, papillary, or tubular patterns. Tumor cells consisted of epithelioid cells, rhabdoid-like cells (approximately 30%), and spindle cells (Fig. 5B and C).

##### 3.1.5.8. Mitotic activity and necrosis

The mitotic count was approximately 3 per 10 HPF, with no tumor necrosis observed.

##### 3.1.5.9. Immunohistochemical expression

**Figure 6.**
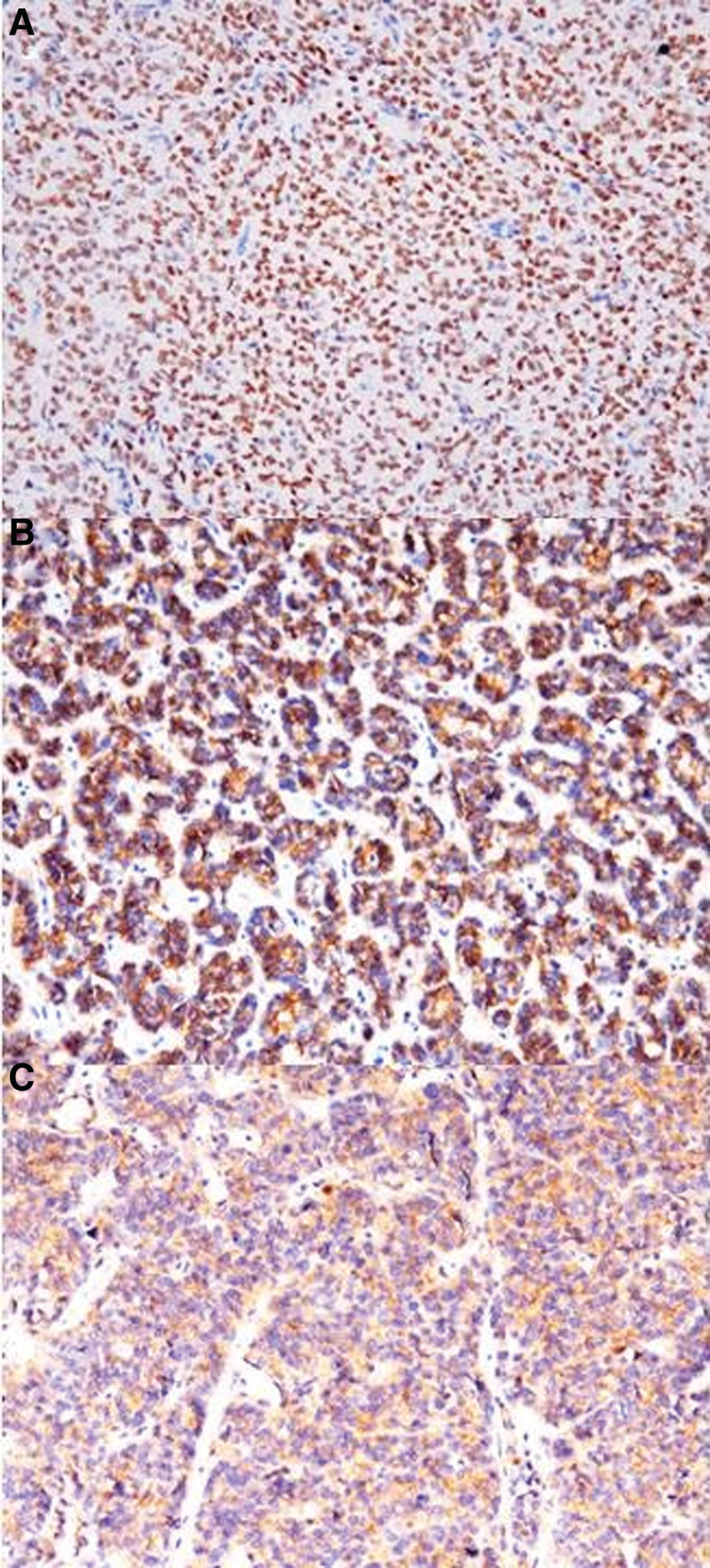
Positivity for WT1 (A), CD56 (B), and CD99 (C).

All 4 cases showed diffuse expression of ER, PR, WT1 (Fig. 6A), as well as desmin. Cases 1, 2, and 3 expressed CD56 and CD99 (Fig. 6B and C). Expression of other epithelial, myogenic, and sex cord markers varied among cases. Notably, none of the cases expressed H-caldesmon or inhibin (Table 1), and all retained expression of INI1, SMARCA2, and SMARCA4.

**Table 1:** Four cases expression.

Case no.	Gene fusion	ER	PR	α-inhibin	Calretinin	CD56	CD99	WT1	CD10	SF1	Desmin	SMA	h-caldesmon	AE1/AE3	EMA	Ki67 (%)
1	ESR1-NCOA2	Strong	Moderate	−	Focal+	+	+	+	Focal+	Focal+	+	−	−	+	Focal+	10
2	ESR1-NCOA2	Strong	Strong	−	Focal+	+	+	+	−	−	+	+	−	−	−	40
3	ESR1-NCOA2	Strong	Moderate	−	−	+	+	+	−	−	+	−	−	−	Focal+	45
4	ESR1-NCOA3	Strong	Moderate	−	−	/	−	+	−	Focal+	+	+	−	Focal+	Focal+	15

+ = positive, − = negative, ER = estrogen receptor, PR = progesterone receptor.

##### 3.1.5.10. FISH expression and next-generation sequencing

**Figure 7.**
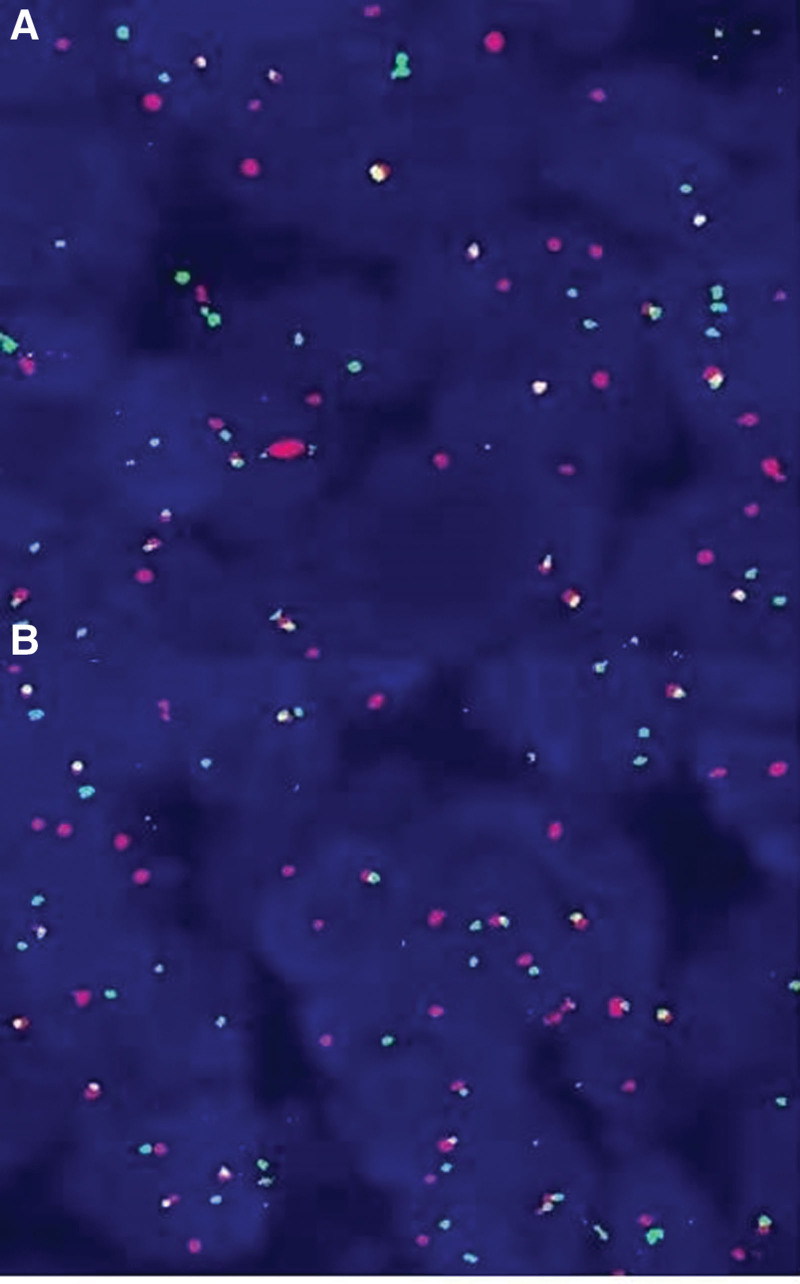
FISH showing break-apart signals for ESR1 (A) and NCOA3 (B). FISH = fluorescence in situ hybridization.

**Figure 8.**
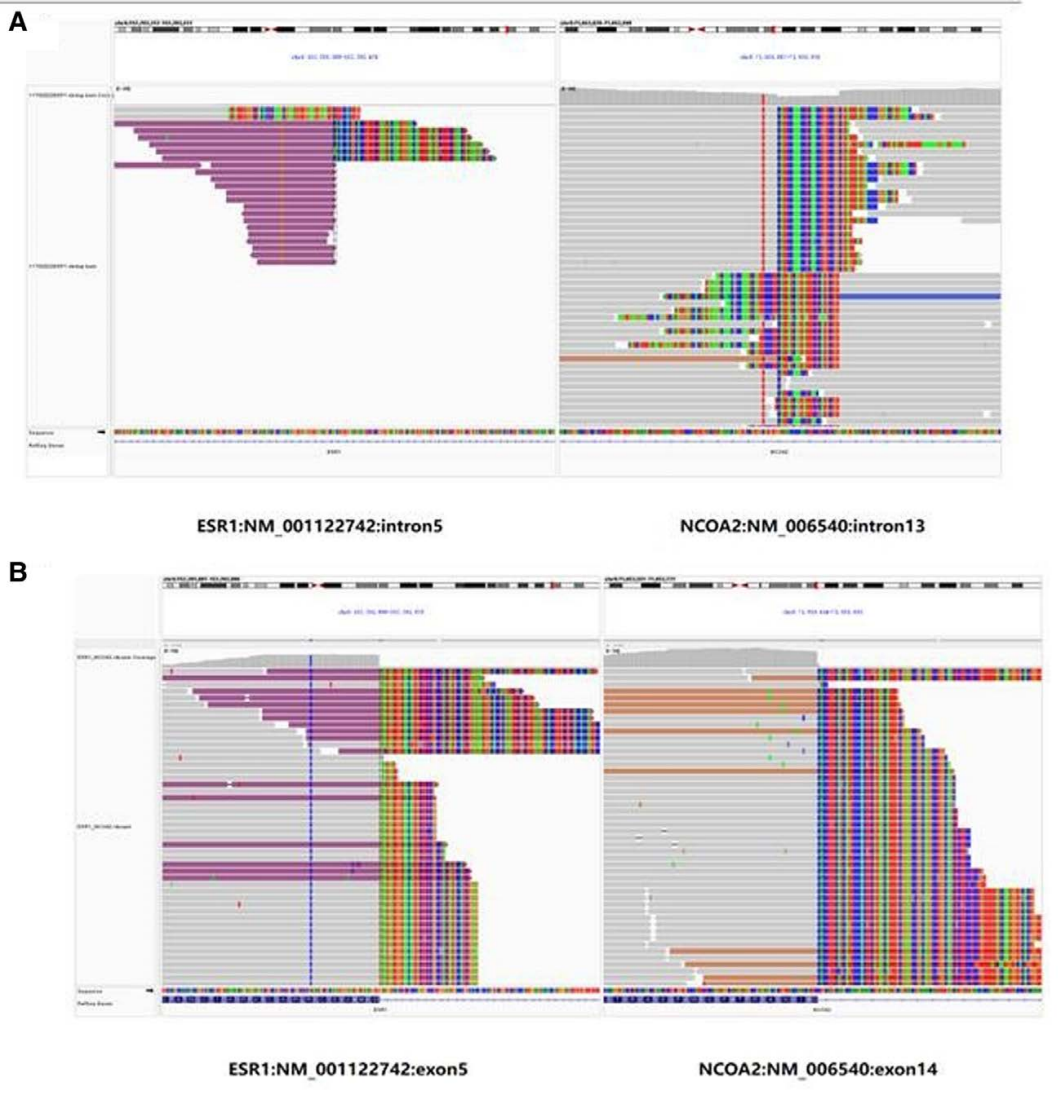
Integrative Genomics Viewer visualized data for case 1 (A) and case 2 (B). For case 1 (A), the 3′ end of intron 5 of ESR1 (left panel) fusing to the 5′ end of intron 13 of NCOA2 (right panel). For case 2 (B), the 3′ end of exon 5 of ESR1 (left panel) fusing to the 5′ end of exon 14 of NCOA2.

FISH analysis detected breaks in ESR1 and NCOA2 in cases 1, 2, and 3, and breaks in ESR1 and NCOA3 in case 4 (Fig. 7). Next-generation sequencing revealed a fusion between intron 5 of ESR1 and intron 13 of NCOA2 in case 1 (Fig. 8A), and a fusion between exon 5 of ESR1 and exon 14 of NCOA2 in case 2 (Fig. 8B).

## 4. Discussion

UTROSCT are rare mesenchymal tumors. Type I tumors typically have an endometrial stromal component with focal sex cord-like elements and often tend to recur and metastasize. Type II tumors are predominantly or exclusively epithelial in appearance, resembling ovarian sex cord tumors, and contain minimal to no stromal component. These tumors typically follow a benign course and rarely recur or metastasize. The term UTROSCT specifically refers to type II tumors. UTROSCTs resemble ovarian sex cord tumors to varying degrees, variably expressing hormone receptors, epithelial, smooth muscle, and sex cord markers, and displaying epithelial and sex cord-like differentiation upon ultrastructural analysis.^[1–7,19]^ In the fifth edition of the World Health Organization Classification of Tumors,^[3]^ UTROSCTs are regarded as tumors of uncertain malignant potential. Although most UTROSCTs are associated with a benign clinical course, some exhibit aggressive behavior with recurrence or metastasis, primarily late recurrence or metastasis.^[1–7]^

The prognosis and criteria for malignancy are not well-established. Clinical outcomes for patients with this type of tumor are mostly reported in the form of case reports and small case series. A recent report indicated that 23.5% of UTROSCTs develop metastases, and 8.8% of patients die from the disease.^[6]^ Cases with malignant behavior were commonly seen in older patients with larger tumors, which are more likely to exhibit necrosis, lymphovascular invasion, cervical involvement, significant nuclear atypia, and increased mitotic activity. However, only necrosis and significant mitotic figures showed statistical significance. The study concluded that there is a significant overlap between benign and malignant UTROSCT morphology, making morphology alone unreliable for assessing prognosis. It suggests considering UTROSCT as a tumor with definite malignant potential.^[1]^ Another larger case series collected 75 cases of UTROSCT. Among 58 patients with follow-up data, 5 had recurrences/metastases, and of these, 2 died from the disease. Compared to benign UTROSCT, malignant UTROSCT had 3 or more of the following 5 features: tumor size >5 cm; at least moderate cytologic atypia;  ≥3 mitoses per 10 HPF; infiltrative borders; necrosis. In this study, one of the 5 malignant UTROSCT cases exhibited extensive rhabdoid morphology. The unique rhabdoid morphologic features include eccentric vesicular nuclei, prominent nucleoli, and abundant eosinophilic cytoplasm with hyaline inclusions, resembling rhabdomyoblasts. The term “rhabdoid” is retained merely to highlight the resemblance to rhabdomyosarcoma.^[20,21]^ It has been proposed that rhabdoid morphology is related to aggressive biological behavior, as evidenced by lymph node metastasis, local infiltration, and early recurrence.^[9,20–22]^ Many tumors can exhibit rhabdoid-like cells, and UTROSCT can also show rhabdoid differentiation. For instance, a study by Jennifer A. Bennett et al examined 3 cases of UTROSCT with malignant behavior that had significant rhabdoid morphology. Similarly, research by Rui Bi et al suggested that rhabdoid features might be a risk factor for recurrence, but this characteristic has received limited attention in the literature.

To investigate the relationship between rhabdoid morphology and the prognosis of UTROSCT, we synthesized data from 37 cases reported in the literature with rhabdoid features and confirmed as UTROSCT^[11,12,23–25]^ (Table 2), as well as 4 cases from our study, totaling 41 cases. Among these, tumor cells exhibited exclusively rhabdoid morphology in 3 cases; in 10 cases, the majority of tumor cells (>50%) had rhabdoid morphology; in 1 case, there was no rhabdoid morphology in intrauterine lesions, but about 20%–25% of extrauterine lesions showed rhabdoid features; in 1 case, approximately 30% of the tumor cells had rhabdoid morphology; in 21 cases, there was a minor presence of rhabdoid morphology; and in 5 cases, rhabdoid morphology was described without mentioning its proportion or providing prognostic information. The results indicated: necrosis and sex cord-like structures appeared in cases with varying proportions of rhabdoid cells, without a corresponding relationship between the proportion of rhabdoid cells and the rate of mitosis; all 3 cases with exclusively rhabdoid morphology experienced distant recurrence or metastasis; of the 10 cases with a majority of rhabdoid morphology, 4 had distant recurrence or metastasis, 1 case had invasion of surrounding tissue and lymph node metastasis, 1 case did not mention prognosis information, and 4 cases had no recurrence or metastasis; the case with 30% rhabdoid tumor cells had a short follow-up period of only 6 months and currently shows no recurrence or metastasis; the case with extrauterine lesions showing rhabdoid morphology involved multiple extrauterine organs, but did not mention information on recurrence or metastasis; of the 21 cases with a minor presence of rhabdoid morphology, 3 experienced distant recurrence or metastasis (1 of the 21 cases lacked prognostic information), suggesting that a higher proportion of rhabdoid morphology is associated with an increased likelihood of distant recurrence or metastasis. Even in cases with only a small amount of rhabdoid morphology, distant recurrence and metastasis occurred, indicating that although rhabdoid morphological changes alone may not predict recurrence or metastasis of UTROSCT, this unusual morphology may closely relate to aggressive behavior and signifies a connection with potential adverse outcomes.

**Table 2:** Rhabdoid differentiation in previously reported UTROSCTs.

References	No. case	Fusion gene	Age (yr)	Size (cm)	Rhabdoid extent	Growth pattern	Necrosis	Mitosis (/10 HPF)	Surgery	Follow-up and treatment	Sex cord marker	Extrauterine disease at dianosis
Bennett et al^[9]^	1	ESR1-NCOA2	37	–	Totally	Sheets, cords and trabeculae, pseudopapillary	Np	PT = 0 RT = 16	TAH	Pelvic recurrence after 7 yr; the second and third operation, pelvic radiation, cisplatin chemotherapy after recurrence	WT1, CD56	–
	2	ESR1-NCOA2	54	–	Totally	Sheets, cords and trabeculae, pseudopapillary	Np	PT = 4 RT = 17	TAHBSO	Pelvic recurrence after 9 yr; chemotherapy after recurrence	WT1, CD56	–
	3	ESR1-NCOA2	30	–	PT = prominent (50%) RT = totally	Cord, trabecular, retiform, pseudopapillary	–	2	TAH	Omental recurrence after 32 yr; a second recurrence after 2 yr; the second and third operation, chemotherapy after recurrence	WT1, CD56, calretinin	–
Devereaux et al^[14]^	4	GTF2A1-NCOA2	42	8.8	Extrauterine sites = 20%–25% PT = 0	Clusters, cords	P	2	TAHBSO	–	Calretinin, inhibin (patchy)	Myometrium, uterine serosa Bilateral ovarian Surfaces, large bowel serosa Anterior abdominal
Yin et al^[7]^	5	GREB1-NCOA2	51	8.5	Occasionally (<5%)	Sheets, fascicles, focally cords	P	3	TAHBSO + NLD	Alive with no disease (12 mo), no chemoradiotherapy	WT1, CD56, calretinin, CD99	–
Boyraz et al^[4]^	6–18	–	–	–	Minor	Diffuse, nested	NP	–	–	Alive with no disease (22–192 mo), NA	–	–
	19	–	–	3.5	Extensive (>50%)	Diffuse, nested	P, focal	4	–	Alive with no disease (24 mo), NA	–	–
	20	–	–	13	Extensive (>50%)	Diffuse, nested	P	7	–	Peritoneum recurrence after 60 mo, alive with disease, NA	–	Extensively involving serosa
	21–25	–	–	–	Present	Diffuse, nested	–	–	–	–	–	–
Hurrell and McCluggage^[15]^	26–28	–	43, 73, 84	0.8, 2.0, 3.0	Prominent	Nests, cords, trabeculae	–	1	TAHBSO	Live with no disease (6–24–132 mo), NA	CD56, WT1, CD99 (focal, case 27, 28), calretinin (case 26, 27 focal, case 28 diffuse), inhibin (focal, case 26, 28)	–
Croce et al^[16]^	29	GREB1-CTNNB1	70	10	Focally	Diffuse, tubular, nested, trabecular	–	1	TAHBSO + NLD	Pelvic recurrence after 17 mo, the second operation; aromatase inhibitors after second surgery, lung metastases and peritoneal recurrence after 29 mo	CD10, calretinin (focal), WT1 (focal)	–
Cheng-Han Lee et al^[17]^	30	GREB1-NCOA2	56	10	Focally	Diffuse, sheets, fascicular	P	8	TAHBSO + NLD	Alive with no disease in 3 wk, ND	CD56, CD99, WT1, calretinin (focal)	–
Goebel et al^[2]^	31	ESR1-NCOA3	29	–	Focally	Nested, sertoliform	NP	2	Myomectomy	–	Inhibin, calretinin, CD10	–
	32	ESR1-NCOA2	20	7.7	Prominent	Nested, corded	NP	6	Myomectomy	–	Inhibin, calretinin, WT1, CD10	–
Rui Bi et al^[8]^	33	GREB1-NCOA2	56	10	Focally	Nested, sex cord, whorled, sertoliform, trabecular	–	PT = 3 RT = 3	TAHBSO	Pelvic recurrence after 30 mo of surgery, alive (65 mo); concurrent paclitaxel and cisplatin chemotherapy after recurrence	WT1, CD99, CD56, inhibin (focal), calretinin (focal)	–
	34	GREB1-NCOA2	38	–	Focally	Nested, diffuse, sex cord	–	PT = – RT = 3	TAHBSO	Pelvic and omentum recurrence after 101 mo, alive with pelvic mass (124 mo); concurrent cisplatin and doxorubicin chemotherapy after the first operation; megestrol, cyclophosphamide, cisplatin and pharmorubicin after recurrence	–	–
	35	ESR1-NCOA2	40	4	Prominent	Cord, sertoliform, trabecular	–	PT < 1 RT < 1	TAHBSO followed by polypectomy twice	Recurrence 21 and 64 mo after twice polypectomy; alive after TAHBSO (109 mo); no chemoradiotherapy	WT1, CD56 (focal), CD10 (focal)	–
	36	GTF2A1-NCOA2	30	6	Focally	Solid area composed of plump spindle to oval cells, vague sex cord pattern, epithelioid cells scattered or in clusters in the myxoid matrix	Patchy necrosis	2	Myomectomy	Alive with no disease (10 mo), no chemoradiotherapy	Calretinin (patchy), CD56, CD99, CD10	–
Koki Ise et al^[18]^	37	Without ESR1-NCOA2/3 and GREB1-NCOA1/2	75	7.4	Focally	Cluters, nests, cords, distinctive myxoid component	–	1–4	TAHBSO + NLD	Alive with no disease (24 mo), no chemoradiotherapy	CD10, CD56, SF1, inhibin, calretinin (focal)	–

As much of the early literature lumped endometrial stromal sarcomas with sex cord-like differentiation with UTROSCTs, cases where a definitive diagnosis of UTROSCT could not be determined based on the available information were excluded.

– = not available/performed, LND = regional lymph node dissection, NA = not available, ND = no disease, NP = not present, P = present, PT = primary tumor, RT = recurrent tumor, TAH = total abdominal hysterectomy, TAHBSO = total abdominal hysterectomy and bilateral salpingo-oophorectomy.

UTROSCT characteristically exhibits a polyphenotypic immunophenotype with the co-expression of epithelial, hormone receptors, smooth muscle markers, and sex cord-stromal markers including inhibin, calretinin, WT1, CD56, CD99, SF1, FOXL2, and Melan-A. The expression of sex cord markers aids in diagnosis and exclusion of other lesions.^[10,12,13,23,25,26]^ In our series, we noted that WT1, CD56, and CD99 were more frequently expressed in UTROSCT with rhabdoid features and are relatively stable sex cord markers. When UTROSCT exhibits predominantly rhabdoid morphology (>50%) with less sex cord differentiation, we recommend prioritizing the use of WT1, CD56, and CD99 as sex cord markers, alongside epithelial, myoid, and hormone receptors to confirm or exclude UTROSCT.

Beyond morphological characteristics, some studies have attempted to establish correlations between the clinicopathological features and genotype (molecular alterations) of UTROSCT. On a molecular level, UTROSCT can have a variety of fusion genes, such as ESR1-NCOA2, ESR1-NCOA3, GREB1-NCOA2, GREB1-NCOA1, GREB1-SS18, GREB1-NR4A3, GREB1-CTNNB1, and GTF2A1-NCOA2. Research results from multiple studies suggest that different fusion genes are associated with distinct morphological changes and prognoses.^[10,12,13,23,25,26]^ For instance, UTROSCT with GREB1 rearrangements tend to present in older patients, with larger tumors, higher stages, more intramural masses, and lack prominent sex cord-like features. The highest recurrence rate in UTROSCT is seen with GREB1::NCOA2 (57%), followed by GREB1::NCOA1 (40%), ESR1::NCOA2 (33%), and ESR1::NCOA3 (14%).

We also sought to establish a correlation between the rhabdoid morphology and molecular changes of UTROSCT. By integrating the literature and our collective 41 cases of UTROSCT with rhabdoid features,^[8,10,12,13,23,25,26]^ we found that all 3 cases where tumor cells exhibited exclusively rhabdoid morphology had ESR1-NCOA2 molecular alterations (all recurred/metastasized). Of the 10 cases where the majority of tumor cells (>50%) had rhabdoid morphology, molecular changes included 5 cases with ESR1-NCOA2 fusions (four recurred/metastasized) and 5 cases without information on molecular changes. One case with no rhabdoid morphology in the intrauterine lesion but approximately 20%–25% rhabdoid morphology in extrauterine lesions had a GTF2A-NCOA2 molecular alteration. One case with approximately 30% rhabdoid cell morphology had an ESR1-NCOA3 fusion. Of the 21 cases with a minor presence of rhabdoid morphology, 13 had no information on molecular alterations, while the remaining 8 included 4 cases with GREB1-NCOA2 (2 recurred/metastasized), 1 with GREB1-CTNNB1 (recurred/metastasized), 1 with ESR1-NCOA3, 1 with GTF2A1-NCOA2, and 1 that showed no fusions of ESR1-NCOA2/3 or GREB1-NCOA1/2. Five cases described rhabdoid morphology without mentioning molecular alteration information.

Our findings suggest that patients with ESR1 and NCOA2/3 rearrangements are prone to exhibit rhabdoid morphology. Both GREB1 and ESR1 fusions can present with rhabdoid morphology, consistent with the findings of Rui Bi et al ESR1 and NCOA2/3 rearrangements combined with significant rhabdoid morphology of tumor cells (>50%) indicate a poorer prognosis, with a higher likelihood of late recurrence and metastasis. Cases with GREB1 rearrangements, even with a minor presence of rhabdoid morphology, experienced recurrence/metastasis, suggesting that GREB1 rearrangements portend a poor prognosis even with focal rhabdoid morphology.

The treatment of UTROSCT currently lacks a standardized approach.^[6–8,21,26]^ Surgery, specifically hysterectomy with or without salpingo-oophorectomy, is the most commonly utilized method. Lymph node dissection may be performed based on the patient’s condition. For patients with fertility preservation needs, fertility-sparing surgery is attempted when possible. The role of adjuvant chemotherapy remains unclear and is only recommended for cases of recurrence or metastasis. Some authors suggest using chemotherapeutic agents such as bleomycin, etoposide, and cisplatin, or considering hormone receptor antagonists/modulators, although their efficacy is not well-established. Due to the relative rarity of UTROSCT, clinicians sometimes resort to treatment regimens similar to those used for uterine endometrial stromal sarcomas in cases of recurrence or metastasis. Literature reports and our collected 41 cases of UTROSCT with rhabdoid features mostly underwent total hysterectomy or total hysterectomy with salpingo-oophorectomy. Among these, 10 cases recurred or metastasized, and 2 cases involved peripheral tissue spread, totaling 12 cases. Some patients underwent 2 to 3 surgeries post-recurrence, along with chemotherapy, radiation therapy, or endocrine therapy, but the reported cases did not assess the efficacy of surgery and subsequent treatments. In our collection of 4 cases, 2 deceased cases (cases 1 and 3) with significant rhabdoid UTROSCT underwent multiple surgeries, hormonal antagonist therapy, chemotherapy, and immunotherapy after the first recurrence. However, the disease continued to progress, resulting in further metastatic spread, a worse clinical course post-recurrence, and poor prognosis. Cases 2 and 4 underwent several rounds of chemotherapy with follow-up times of 6 to 12 months and have not shown recurrence or metastasis to date. Therefore, whether routine chemotherapy is necessary after diagnosis for UTROSCT with extensive rhabdoid morphology (>50%) and which treatment strategies should be adopted after recurrence or metastasis still require further case accumulation.

The strengths of this study lie first in its multidisciplinary approach, as it involves collaboration between clinicians, pathologists, molecular biologists, and other fields to comprehensively analyze cases of UTROSCT with rhabdoid features from different perspectives, providing important insights for a deeper understanding of the disease. Second, by employing molecular biology techniques such as FISH and NGS, the authors identified molecular alterations associated with the rhabdoid features of UTROSCT, offering clues for further research into the pathogenesis of the disease.

However, the study also has some limitations. First, the sample source is limited: the cases studied mainly come from 1 hospital, which may introduce sample selection bias, affecting the representativeness and generalizability of the results. Second, there is a lack of systematic evaluation of treatment outcomes: although the use of different treatment modalities is described, there is a lack of systematic evaluation of treatment outcomes, making it difficult to determine which treatment strategy is more effective for cases of UTROSCT with rhabdoid features. Finally, prognosis analysis is limited: despite the analysis of clinical features and molecular changes in the cases, the analysis of the prognostic impact of UTROSCT with rhabdoid features is restricted due to the limited number of cases from a single institution, necessitating further large-scale, multicenter studies to validate the findings.

In summary, this study focuses on the prognosis, molecular alterations, and clinical treatment strategies of UTROSCT with rhabdoid morphology. UTROSCT with extensive or significant rhabdoid features is closely related to aggressive behavior, with a higher likelihood of distant recurrence or metastasis, or presenting with malignant biological behavior at initial diagnosis. UTROSCT with ESR1 and NCOA2/3 rearrangements is more likely to exhibit rhabdoid morphology, often with a substantial proportion of rhabdoid cells. UTROSCT with significant rhabdoid morphology and ESR1 or NCOA2/3 rearrangements, as well as UTROSCT with rhabdoid morphology and GREB1 rearrangements, require long-term follow-up.

We believe that for UTROSCT cases with rhabdoid morphology, it is necessary to report the proportion of such cells in detail in the pathological report, enhance molecular testing, ensure accurate tumor staging, and manage the disease course over the long term. Limited cases indicate that patients with significant rhabdoid features may still show progression of the tumor despite various combined clinical treatments post-recurrence. Therefore, more patients, more comprehensive clinical data, and longer follow-up times are needed to further study the molecular changes, prognosis, and treatment strategies for UTROSCT with rhabdoid morphology, especially treatment strategies post-recurrence.

## 5. Conclusion

This study highlights the critical importance of detailed pathological reporting, comprehensive molecular testing, and thorough tumor staging in managing UTROSCT with rhabdoid features. Our case series of 4 patients demonstrates that UTROSCT with significant rhabdoid morphology is closely associated with aggressive clinical behavior, including a higher propensity for recurrence and metastasis. The presence of ESR1 and NCOA2/3 rearrangements was consistently observed in tumors with extensive rhabdoid differentiation, suggesting a molecular basis for the aggressive nature of these tumors.

## Acknowledgments

The authors thank OrigiMed Technology Inc., Shanghai, for performing the NGS based on DNA/RNA.

## Author contributions

**Data curation:** Hongling Li, Jinhui Zhang, Xingyan Wu, Zengwei Chen, Rongjun Mao.

**Formal analysis:** Hongling Li, Le Xie, Jinhui Zhang, Xingyan Wu, Rongjun Mao.

**Funding acquisition:** Hongling Li, Le Xie, Jinhui Zhang, Zengwei Chen, Rongjun Mao.

**Writing—original draft:** Hongling Li, Le Xie, Yuanyuan Xu, Xingyan Wu, Zengwei Chen, Rongjun Mao.

**Writing—review & editing:** Hongling Li, Jinhui Zhang, Rongjun Mao.

**Conceptualization:** Le Xie, Jinhui Zhang, Yuanyuan Xu, Xingyan Wu, Zengwei Chen, Rongjun Mao.

**Investigation:** Le Xie, Yuanyuan Xu.

## References

[1] Boyraz B Watkins JC Young RH Oliva E. Uterine tumors resembling ovarian sex cord tumors: a clinicopathologic study of 75 cases emphasizing features predicting adverse outcome and differential diagnosis. Am J Surg Pathol. 2023;47:234–47. 36306239 10.1097/PAS.0000000000001981

[2] Goebel EA Hernandez Bonilla S Dong F. Uterine tumor resembling ovarian sex cord tumor (UTROSCT): a morphologic and molecular study of 26 cases confirms recurrent NCOA1-3 rearrangement. Am J Surg Pathol. 2020;44:30–42. 31464709 10.1097/PAS.0000000000001348 PMC8223168

[3] Höhn AK Brambs CE Hiller GGR May D Schmoeckel E Horn LC. 2020 WHO classification of female genital tumors. Geburtshilfe Frauenheilkd. 2021;81:1145–53. 34629493 10.1055/a-1545-4279 PMC8494521

[4] Kaur K Rajeshwari M Gurung N. Uterine tumor resembling ovarian sex cord tumor: a series of six cases displaying varied histopathological patterns and clinical profiles. Indian J Pathol Microbiol. 2020;63(Supplement):S81–6. 32108635 10.4103/IJPM.IJPM_340_19

[5] Bi R Yao Q Ji G. Uterine tumor resembling ovarian sex cord tumors: 23 cases indicating molecular heterogeneity with variable biological behavior. Am J Surg Pathol. 2023;47:739–55. 37132508 10.1097/PAS.0000000000002046

[6] Moore M McCluggage WG. Uterine tumour resembling ovarian sex cord tumour: first report of a large series with follow-up. Histopathology. 2017;71:751–9. 28656712 10.1111/his.13296

[7] Yin X Wang M He H Ru G Zhao M. Uterine tumor resembling ovarian sex cord tumor with aggressive histologic features harboring a GREB1-NCOA2 fusion: case report with a brief review. Int J Gynecol Pathol. 2023;42:54–62. 35081070 10.1097/PGP.0000000000000849

[8] Dickson BC Childs TJ Colgan TJ. Uterine tumor resembling ovarian sex cord tumor: a distinct entity characterized by recurrent NCOA2/3 gene fusions. Am J Surg Pathol. 2019;43:178–86. 30273195 10.1097/PAS.0000000000001153 PMC6719705

[9] Folpe AL Graham RP Martinez A Schembri-Wismayer D Boland J Fritchie KJ. Mesenchymal chondrosarcomas showing immunohistochemical evidence of rhabdomyoblastic differentiation: a potential diagnostic pitfall. Hum Pathol. 2018;77:28–34. 29559236 10.1016/j.humpath.2018.03.012

[10] Grither WR Dickson BC Fuh KC Hagemann IS. Detection of a somatic GREB1-NCOA1 gene fusion in a uterine tumor resembling ovarian sex cord tumor (UTROSCT). Gynecol Oncol Rep. 2020;34:100636. 32964092 10.1016/j.gore.2020.100636 PMC7490989

[11] Hurrell DP McCluggage WG. Uterine tumour resembling ovarian sex cord tumour is an immunohistochemically polyphenotypic neoplasm which exhibits coexpression of epithelial, myoid and sex cord markers. J Clin Pathol. 2007;60:1148–54. 17182656 10.1136/jcp.2006.044842 PMC2014850

[12] Ise K Tanei ZI Oda Y. A case of uterine tumor resembling ovarian sex cord tumor with prominent myxoid features. Int J Gynecol Pathol. 2024;43:41–6. 37406360 10.1097/PGP.0000000000000949

[13] Kertowidjojo EC Bennett JA. Update on uterine mesenchymal neoplasms. Surg Pathol Clin. 2022;15:315–40. 35715164 10.1016/j.path.2022.02.008

[14] Qijun C Wei W Cheng W Dongni L. Clinicopathological features and molecular genetic changes in 17 cases of uterine tumor resembling ovarian sex cord tumor. Hum Pathol. 2024;143:33–41. 38000680 10.1016/j.humpath.2023.11.007

[15] Shibahara M Kurita T Harada H Tsuda Y Hisaoka M Yoshino K. Therapeutic management of uterine tumours resembling ovarian sex cord tumours including a focus on fertility: a systematic review. Eur J Obstet Gynecol Reprod Biol. 2024;295:1–7. 38310674 10.1016/j.ejogrb.2024.01.039

[16] Watrowski R Palumbo M Guerra S. Uterine tumors resembling ovarian sex cord tumors (UTROSCTs): a scoping review of 511 cases, including 2 new cases. Medicina (Kaunas). 2024;60:179. 38276058 10.3390/medicina60010179 PMC10820159

[17] Haas BJ Dobin A Li B Stransky N Pochet N Regev A. Accuracy assessment of fusion transcript detection via read-mapping and de novo fusion transcript assembly-based methods. Genome Biol. 2019;20:213. 31639029 10.1186/s13059-019-1842-9 PMC6802306

[18] Moes-Sosnowska J Skupinska M Lechowicz U. FGFR1-4 RNA-based gene alteration and expression analysis in squamous non-small cell lung cancer. Int J Mol Sci. 2022;23:10506. 36142417 10.3390/ijms231810506 PMC9505002

[19] Lin J Liu L Wang L. The management of uterine tumor resembling an ovarian sex cord tumor (UTROSCT): case series and literature review. World J Surg Oncol. 2024;22:42. 38310233 10.1186/s12957-024-03319-3 PMC10837875

[20] Al-Hussaini M Hirschowitz L McCluggage WG. Uterine neoplasms composed of rhabdoid cells do not exhibit loss of INI1 immunoreactivity and are not related to childhood malignant rhabdoid tumor. Int J Gynecol Pathol. 2008;27:236–42. 18317218 10.1097/PGP.0b013e31815aca67

[21] Miyazaki T Aishima S Fujino M. Neuroendocrine tumor of the pancreas with rhabdoid feature. Virchows Arch. 2018;473:247–52. 29938394 10.1007/s00428-018-2398-x PMC6096768

[22] Klimis T Karvounis H. Renal cell carcinoma with rhabdoid features. Divergent differentiation of conventional (clear cell) carcinoma. J BUON. 2008;13:433–6. 18979563

[23] Croce S Lesluyes T Delespaul L. GREB1-CTNNB1 fusion transcript detected by RNA-sequencing in a uterine tumor resembling ovarian sex cord tumor (UTROSCT): a novel CTNNB1 rearrangement. Genes Chromosomes Cancer. 2019;58:155–63. 30350331 10.1002/gcc.22694

[24] Devereaux KA Kertowidjojo E Natale K Ewalt MD Soslow RA Hodgson A. GTF2A1-NCOA2-associated uterine tumor resembling ovarian sex cord tumor (UTROSCT) shows focal rhabdoid morphology and aggressive behavior. Am J Surg Pathol. 2021;45:1725–8. 34334688 10.1097/PAS.0000000000001786 PMC8585683

[25] Lee CH Kao YC Lee WR. Clinicopathologic characterization of GREB1-rearranged uterine sarcomas with variable sex-cord differentiation. Am J Surg Pathol. 2019;43:928–42. 31094921 10.1097/PAS.0000000000001265

[26] Ye S Wu J Yao L He J. Clinicopathological characteristics and genetic variations of uterine tumours resembling ovarian sex cord tumours. J Clin Pathol. 2022;75:776–81. 34348985 10.1136/jclinpath-2021-207441 PMC9606539

